# Neural correlates of subjective cognitive decline in adults at high risk for Alzheimer’s disease

**DOI:** 10.3389/fnagi.2023.1067196

**Published:** 2023-02-01

**Authors:** Liat Ben-Ami, Ramit Ravona-Springer, Galia Tsarfaty, Reut Raizman, Aleeza Shumacher, Inbal Sharvit-Ginon, Lior Greenbaum, Barbara B. Bendlin, Eitan Okun, Anthony Heymann, Michal Schnaider Beeri, Abigail Livny

**Affiliations:** ^1^Division of Diagnostic Imaging, Sheba Medical Center, Ramat Gan, Israel; ^2^The Leslie and Susan Gonda Multidisciplinary Brain Research Center, Bar-Ilan University, Ramat Gan, Israel; ^3^The Joseph Sagol Neuroscience Center, Sheba Medical Center, Tel HaShomer, Israel; ^4^Memory Clinic, Sheba Medical Center, Ramat Gan, Israel; ^5^Sackler Faculty of Medicine, Tel Aviv University, Tel Aviv, Israel; ^6^Department of Psychology, Bar-Ilan University, Ramat Gan, Israel; ^7^The Danek Gertner Institute of Human Genetics, Sheba Medical Center, Ramat Gan, Israel; ^8^Wisconsin Alzheimer’s Disease Research Center, School of Medicine and Public Health, University of Wisconsin–Madison, Madison, WI, United States; ^9^The Mina and Everard Goodman Faculty of Life Sciences, Bar-Ilan University, Ramat Gan, Israel; ^10^The Paul Feder Laboratory on Alzheimer’s Disease Research, Bar-Ilan University, Ramat Gan, Israel; ^11^Maccabi Healthcare Services, Tel Aviv, Israel; ^12^Department of Psychiatry, Icahn School of Medicine at Mount Sinai, New York, NY, United States; ^13^The Sagol School of Neuroscience, Tel Aviv University, Tel Aviv, Israel

**Keywords:** subjective cognitive decline (SCD), Alzheimer’s dementia (AD), neuroimaging, fMRI, working memory, diffusion tensor imaging, family history (FH), *APOE*

## Abstract

**Introduction:**

Recently, interest has emerged in subjective cognitive decline (SCD) as a potential precursor to Alzheimer’s disease (AD) dementia. Whether individuals with SCD harbor brain alterations in midlife, when AD-related pathology begins, is yet to be elucidated. Furthermore, the role of apolipoprotein ε4 (*APOE* ε*4*) allele, a robust AD risk factor, in the relationship between SCD and brain alterations is unknown. We examined whether *APOE* genotype modulates the association of SCD with brain measures in individuals at high AD risk.

**Methods:**

Middle-aged adults with parental history of AD dementia underwent magnetic resonance imaging (MRI) and the Memory Functioning Questionnaire. Regression analysis tested the extent to which SCD was associated with activation during an functional MRI (fMRI) working-memory task, and white-matter microstructure. *APOE* ε*4* genotype was tested as a moderator.

**Results:**

Among *APOE* ε*4* carriers, but not among non-carriers, SCD was associated with higher activation in the anterior cingulate (*p* = 0.003), inferior, middle, and superior frontal cortices (*p* = 0.041, *p* = 0.048, *p* = 0.037, respectively); and with lower fractional anisotropy in the uncinate fasciculus (*p* = 0.002), adjusting for age, sex, and education.

**Conclusion:**

In middle aged, cognitively normal individuals at high AD risk, higher SCD was associated with greater brain alterations possibly reflecting incipient AD pathology. When accompanied by a family history of AD and an *APOE* ε*4* allele, SCD may have important clinical value, allowing a window for early intervention and for participants’ stratification in AD prevention clinical trials.

## Introduction

Alzheimer’s disease (AD), the leading cause of dementia, accounts for over 50% of all dementia cases worldwide. Due to the expected substantial increase in dementia prevalence in the years to come (Alzheimer’s Association, 2022) and with the recent trial reporting less clinical decline using a novel therapeutic agent (van Dyck et al., 2022), extensive scientific effort has and should be directed toward detection of early biomarkers and dementia prevention (Smith and Yaffe, 2014). This is especially relevant in the context of midlife, when the neuropathology of AD begins to aggregate (Racine et al., 2016). In order to implement such intervention strategies effectively, it is crucial to characterize the potential impact of parental family history of AD (FH) and carrying the apolipoprotein ε4 allele (*APOE* ε*4*), two major risk factors for AD which are associated with more rapid cognitive decline and earlier age of onset of AD (Martinez et al., 1998), on brain pathology.

In recent years, an increasing interest has emerged in subjective cognitive decline (SCD) leading to a conceptual framework, by the working group of the Subjective Cognitive Decline Initiative (SCD-I), aimed to increase comparability of research on SCD across settings and studies (Jessen et al., 2014).

Subjective cognitive decline represents an individual’s subjective perception of decline from previous levels of cognitive functioning, while these subtle and early changes cannot be detected on objective neuropsychological tests (Burmester et al., 2016). SCD has been associated with objective memory deficits (Amariglio et al., 2011), future cognitive decline (Jorm et al., 2001), increased risk of clinical conversion to mild cognitive impairment (MCI), dementia and AD (Jungwirth et al., 2008; Neto and Nitrini, 2016). Some studies did not find such associations (Jungwirth et al., 2004; Cargin et al., 2008). If SCD indeed indicates an early disease process, early neuropathological correlates could include alterations to the neural architecture supporting cognitive function, including changes in white matter integrity or in neural function.

As the conceptual SCD framework requires additional validation to serve as a standardized indicator for biomarker-based preclinical AD (Jessen et al., 2014), we propose a neuroimaging perspective of SCD, while applying the framework’s criteria in our study.

Neuroimaging methods have great potential for identifying the underlying pathological processes associated with SCD. Overall, the literature reveals that individuals with SCD show vulnerability of AD related regions. Nonetheless, literature exploring brain activation using task-based functional magnetic resonance imaging (fMRI), and white-matter integrity with diffusion tensor imaging in individuals with SCD reported inconsistent findings (Wang et al., 2020). Older adults with SCD showed increased brain activation relative to controls in frontal, temporal, and parietal regions (Rodda et al., 2009; Dumas et al., 2013; Hu et al., 2017) and decreased brain activation in temporal regions (Erk et al., 2011; Hayes et al., 2017) while engaged in various cognitive tasks. Participants with SCD also exhibited lower white matter integrity in temporal structures (Li et al., 2016; Ryu et al., 2017). Moreover, the integrity of some white matter tracts, among people with SCD were similar to those of patients with MCI (Hong et al., 2015). Other studies did not report findings in diffusion metrics among these participants (Kiuchi et al., 2014; Viviano et al., 2019).

Only few studies have examined the neural correlates of SCD in individuals at high AD risk due to FH and *APOE* ε*4*. In cognitively unimpaired individuals with FH, SCD was associated with a brain connectivity pattern that is similar to that of early AD (Verfaillie et al., 2018). *APOE* ε*4* carriers with SCD also show higher tau and higher amyloid deposition, defining features of AD (Risacher et al., 2016); reduced gray matter volume in temporal, parietal, and frontal areas (Lee et al., 2016); and reduced whole-brain white matter volume (Dauphinot et al., 2020). In a previous study conducted by our group, SCD was correlated with significant cortical thinning in temporal, paralimbic, and parietal regions, even after adjusting for FH and *APOE* ε*4* (Schultz et al., 2015).

To date, associations of SCD with brain structure and function have mostly focused on old age rather than in midlife, a critical period for potential interventions. Thus, whether SCD in midlife is related to neurobiological changes, remains to be elucidated, especially among high-risk individuals for whom SCD may reflect the initial process of neurodegeneration. To determine the role of SCD in high AD risk middle-aged individuals, we tested whether *APOE* genotype modulates the associations of SCD with structural and functional brain measures related to cognitive decline as indexed by brain activation during an fMRI working memory task and white matter integrity, respectively. We hypothesized that associations between SCD and brain indices will be stronger in *APOE* ε*4* carriers compared to *APOE* ε*4* non-carriers.

## Materials and methods

### Participants

Participants were recruited from the Israel Registry for Alzheimer’s Prevention (IRAP), a collaboration between the Sheba Medical Center and Maccabi Health Services (MHS) examining the effect of sociodemographic, cognitive, health-related, lifestyle, laboratory, and genetic factors on cognitive functioning and decline along with neural characteristics in middle-aged offspring of AD patients. The IRAP study design has been previously described in detail (Ravona-Springer et al., 2020). In short, participants’ inclusion criteria was MHS membership, age at enrollment 40–65 years, fluency in Hebrew, with parental history of AD. Medical records of parents of individuals who approach the study team are provided to the study team and a Dementia Questionnaire is administered to potential participants. All the medical history and diagnostic workup available is reviewed together with the Dementia Questionnaire in order to reach a probable AD diagnosis (according to NINCDS-ADRDA criteria). Offspring of probands with partial information about dementia type or with dementia other than AD, are excluded from the study. In addition to a parental family history of AD (an inclusion criterion for the IRAP study), eligibility criteria for this analysis included: (1) available MRI scan; (2) completion of the Memory Functioning Questionnaire (MFQ); (3) available *APOE* genotype.

We have excluded participants with a life threatening disease such as advanced cancer or severe psychiatric disorders such as schizophrenia. Participants were recruited through an advertisement posted on the home-page of the MHS website and through participants’ word of mouth. Briefly, participants completed blood pressure measurements, neuropsychological testing, laboratory testing, *APOE* genotyping, history and current health assessment, lifestyle, dietary habits, medications, depression, anxiety, and a questionnaire on subjective cognitive complaints.

**FIGURE 1:**
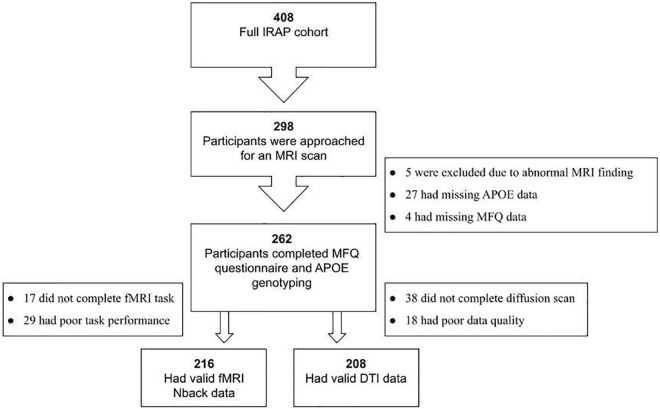
Flowchart of the study cohort. IRA*P*, Israel Registry for Alzheimer’s Prevention; FH, parental family history of AD; MRI, magnetic resonance imaging; MFQ, Memory Functioning Questionnaire; *APOE*, apolipoprotein E gene; fMRI, functional MRI; DTI, diffusion tensor imaging.

The full IRAP study included 408 participants with a parental family history of AD, of whom 298 underwent an MRI scan. Figure 1 provides a flow chart leading to the final Ns of this study. The MRI cohort included 216 participants with valid fMRI data, and 208 with valid DTI data.

### Subjective cognitive decline

Subjective cognitive decline was assessed using the MFQ, a well-validated self-reported measure with four subscales identified by factor analysis (Gilewski et al., 1990). For this study, the 18-item frequency of forgetting (FF) subscale was used as the measure of SCD, as it has been shown to correspond to memory performance (Zelinski et al., 1990), and has been linked to amyloid burden and other significant memory decline measures (Merrill et al., 2012; Snitz et al., 2015; Innes et al., 2017; Sharma et al., 2021) and is known as the most reliable subscale of the MFQ (Amariglio et al., 2012). In this subscale, participants are asked to rate “How often do the following aspects of memory present a problem for you…” on a 7-point Likert scale (1 = “Always,” 7 = “Never”) for different aspects of memory (e.g., memory for faces, names, appointments, etc.). Ratings were summed across 18 items comprising the total FF score. In order to simplify the interpretation of the results, for each participant, the FF scale has been transposed to the opposite direction by subtracting the FF score from the maximal score, so that higher scores correspond to a greater frequency of memory complaints.

### *APOE* ε*4* assessment

Blood samples were taken for DNA. DNA was extracted and frozen in −80°C and so far has been used for APOE genotyping. APOE status is determined based on rs429358 and rs7412 SNPs genotypes (Lim et al., 2019) at LGC company^1^ using Kompetitive allele specific PCR (KASP) technology (Cannon-Albright et al., 2019). Participants who exhibited at least one allele of APOE ε4 (APOE ε34 or APOE ε44) were categorized as APOE ε4 carriers, while those who exhibit no allele of APOE ε4 (APOE ε22, ε23 and ε33) where categorized as APOE ε4 non-carriers (Ravona-Springer et al., 2020).

### Cognitive assessment

Participants completed a comprehensive neuropsychological battery, which was summarized into four domains of episodic memory, executive functions, language, and working memory (for detailed descriptions refer to Ravona-Springer et al., 2020). The episodic memory factor was calculated as the sum of the *z*-scores of the Rey Auditory Verbal Learning Test immediate recall, delayed recall, and recognition scores; and was used as a covariate in secondary analyses.

### Depression assessment

Depressive symptomatology was assessed using the Center for Epidemiologic Studies Depression Scale (CES-D) (Radloff, 1977). The scale consists of 20 Likert-scale questions (scored 0–3) ranging from “rarely/none of the time” to “most/all of the time” about the frequency of depressive symptoms over the past week. The scale of four questions (good, future, happy, and enjoy) was reversed, and all answers were summed across the 20 items to get a total depression score.

### MRI data acquisition

Magnetic resonance imaging scans were acquired at the diagnostic imaging division, Sheba Medical Center, on a 3 Tesla whole body MRI scanner (GE Signa HDxt). For registration purposes high-resolution images (1 mm^3^) were acquired, using a standard 3D inversion recovery prepared fast gradient echo pulse (FSPGR) T1-weighted sequence: TR = 8.85 s, TE = 3.5 s, flip angle = 12°, TI = 450 ms, matrix 256 × 256, field of view (FOV) 25.6 × 25.6 cm. Spin-echo diffusion weighted echo-planar imaging (EPI) sequence was performed as well: 39 axial slices, 1 × 1 × 4 mm resolution, TR = 10 s, TE = 91.5 ms, matrix 128 × 128. We acquired 25 diffusion weighted images in isotropically distributed directions, with *b* = 1,000 s/mm^2^ (Δ/δ = 33/26 ms) and an additional non-DWI image (b0). A T2*-weighted gradient-echo EPI sequence was also acquired: TR = 3 s, TE = 32 ms, flip angle = 90°, matrix 64 × 64, FOV 22 × 22 cm, 40 contiguous oblique axial slices, slice width 3 and 0.4 mm gap.

### fMRI working memory Nback task

The Nback task is a widely used task in fMRI studies for evaluating working memory (Owen et al., 2005). In this task, for each stimulus in a continuous series, participants indicate whether the item matches a stimulus presented “n” stimuli previously. Our task used letter stimuli presented at two different memory load conditions (1-, 2-back; for details see Livny et al., 2018). The Nback paradigm was presented using E-prime 2.0 software (Psychology Software Tools, Inc.), back-projected by a radio-frequency shielded projector system and viewed through a mirror device. Responses were recorded using a Lumina response box (Lumina, Cedrus Corporation, CA, USA). Participants were considered to have reached inclusion criterion if their performance was at least 65% correct responses.

### fMRI Nback task analysis

The fMRI data was processed using Statistical Parametric Mapping software (SPM12, Wellcome Trust Centre for Neuroimaging, London, UK) implemented in MATLAB (The MathWorks Inc., MA, USA). Preprocessing of the functional images included common pipeline preprocessing steps. The pre-processing of functional images included reorienting to the anterior commissure, realignment to the first image using affine transformation, co-registration to the individual’s 3D T1 images, normalization to the stereotactic space of the MNI using a 12-parameter linear affine transformation and smoothing with a Gaussian kernel of 8 × 8 × 8 mm in order to minimize anatomical differences and increase signal to noise ratio (Ashburner, 2009). The preprocessed data was then analyzed individually for each subject using the general linear model (GLM) convolved with the hemodynamic response function.

We produced a map of high working memory load constructed as the contrast of 2-back minus 0-back conditions.

### DTI analysis

Diffusion-weighted images were processed and analyzed using ExploreDTI v4.8.6 software implemented in MATLAB (The MathWorks Inc., MA, USA). Analysis steps included: regularization, brain extraction, eddy current, and motion correction. The diffusion tensors were then calculated using a non-linear regression procedure. Fractional anisotropy (FA) and mean diffusivity (MD) maps were computed, normalized to an MNI space FA template, and smoothed with a full-width half-maximum Gaussian kernel of 5 × 5 × 5 mm, using SPM12 (Wellcome Trust Centre for Neuroimaging, London, UK).

### Region of interest selection

For both structural and functional imaging modalities analyses, a region of interest approach was adopted, allowing us to focus on *a priori* brain regions known to be affected in AD (Yetkin et al., 2006). For fMRI, six cortical regions of interest (ROIs) were extracted from the high working memory load contrast maps: inferior, middle, and superior frontal gyri, anterior cingulate, inferior parietal lobule, and medial temporal lobe. ROIs were identified with the Human Automated Anatomical Labeling (AAL) atlas (Tzourio-Mazoyer et al., 2002) within the WFU Pickatlas tool (V1.2), and signal intensity of the high working memory load contrast was extracted using the MarsBaR toolbox, implemented in SPM8 on MATLAB (The MathWorks Inc., MA, USA). For DTI, data from five white matter ROIs, known to be affected in AD (Alves et al., 2012) were extracted in MNI space, using an in-house matlab script, from the FA and MD maps: genu of the corpus callosum, fornix, the cingulum bundles adjacent to the cingulate cortex, superior longitudinal fasciculi and the uncinate fasciculi. White matter tract ROIs were derived from the JHU ICBM-DTI-81 WM Atlas (Mori et al., 2008), after examining registration between the FA ROIs and white matter tracts. Values at each voxel within an ROI were averaged to obtain a mean value for each ROI. fMRI raw signal was extracted from the left and right homologous regions and was averaged across both sides. DTI signal was thresholded for above 0.2. Finally, subsequent ROI data was transferred to SPSS for further statistical analysis.

### Statistical analyses

For all analyses, the normality of the GLM was verified by inspection of whether the residuals of the regression were normally distributed around zero and had a fixed standard deviation. All analyses were found valid in terms of model normality. Sociodemographic characteristics, depressive symptoms, objective cognitive performance, and SCD score of the two *APOE* genotype groups were compared using Student’s *t*-test.

Associations between our SCD indicator (measured by the FF scale) and sociodemographic characteristics, depression score, and objective cognitive performance, were calculated by Pearson’s correlations test.

The relationship between SCD and brain measurements (fMRI brain activation or FA/MD values) was assessed using a multiple linear regression analysis. The interaction of *APOE* genotype with SCD on brain measurements was examined. To describe the significant interactions, the SCD and brain measurement values were then entered into a partial correlation analysis stratified by *APOE* genotype (*APOE* ε*4* allele carriers and non-carriers). All analyses were controlled for age, sex, and years of education.

We performed a series of additional secondary analyses. First, we examined the interaction of *APOE* genotype with SCD and brain measurements using the total score of the MFQ. In addition, it has been previously argued that SCD is linked to depression (Jorm et al., 2004). Thus, to ensure that our initial findings were not driven by elevated depressive symptoms, we additionally adjusted for the CES-D score.

Third, to assure that our significant results were not driven by variability of objective memory performance, we repeated the GLM interaction analyses, including episodic memory as an additional covariate in the model. Fourth, we have also adjusted for executive function as one of the brain measures in the model, i.e., the Nback task measures working memory and executive function. In addition, our sample included 15 sibling pairs. We thus repeated the GLM interaction analyses again after excluding one random sibling from each pair. Finally, our sample included seven participants with APOE e2/e4 alleles. We thus repeated all analyses again after excluding these participants as well, since the APOE ε2 allele is considered low risk for AD (Strittmatter and Roses, 1996).

A *p*-value of 0.05 was used to determine statistical significance. Statistical analyses were calculated using IBM SPSS Statistics for Windows (version 19; IBM, Armonk, NY, USA).

## Results

Participants (*n* = 262) had a mean age of 54.30 (SD = 6.77) years, range 40–65 years. They were relatively educated with 16.58 (2.94) years of education, all participants were cognitively normal based on cognitive norms for age, sex, and education and had normal episodic memory performance [mean (SD) = 0.039 (0.855)]. APOE ε4 (33.21%) carriers did not differ from non-carriers (66.79%) on age, sex, years of education, depression score, overall cognition, working memory, episodic memory, executive motor, and SCD measures (Table 1).

**TABLE 1:** Characteristics of *APOE* ε*4* carriers vs. *APOE* ε*4* non-carriers.

	*APOE* ε *4* carriers (*n* = 87) (ε2/ε4: *n* = 7; ε3/ε4: *n* = 75; ε4/ε4: *n* = 5)	*APOE* ε *4* non-carriers (*n* = 175) (ε2/ε2: *n* = 1; ε2/ε3: *n* = 15; ε3/ε3: *n* = 159)	*P*-Value
Age	53.81 (6.83) 39–65	54.54 (6.75) 40–66	0.411
Education (years)	16.77 (2.96) 12–26	16.49 (2.94) 11–27	0.481
Female, *n* (%)	57 (65.52)	106 (60.57)	0.499
CES-D	12.10 (8.40) 0–47	11.08 (8.07) 0–45	0.351
Overall cognition	−0.049 (0.47) −1.46 to 0.86	0.03 (0.49) −1.42 to 0.97	0.178
Working memory	−0.06 (0.90) −2.06 to 1.61	0.005 (0.79) −1.67 to 2.44	0.512
Episodic memory	0.009 (0.90) −2.12 to 1.72	0.054 (0.83) −2.74 to 1.64	0.691
Executive motor	0.019 (0.41) −1.12 to 0.81	0.005 (0.47) −1.27 to 1.73	0.811
Language	−0.165 (0.74) −3.48 to 1.16	0.082 (0.74) −3.42 to 2.71	0.012
MFQ_total	242.51 (39.33) 145–322	236.3 (38.22) 130–338	0.224
MFQ_FF	22.52 (13.99) 0–70	23.65 (15) 0–78	0.556

Data presented as mean (SD), range. *APOE* ε*4*, apolipoprotein E, alleles ε34 or ε44; CES-D, Center for Epidemiologic Studies Depression Scale; MFQ_FF, frequency of forgetting scale of the Memory Functioning Questionnaire.

No significant associations were found between SCD and the demographic and cognitive measures, except for the depression score, where we found that higher scores in SCD were associated with greater depression symptoms (*r* = 0.259, *p* = 0.001; Table 2), as was previously described in the literature (Jorm et al., 2004; Montejo et al., 2011).

**TABLE 2:** Association between SCD (MFQ_FF) and objective cognitive performance and demographic characteristics.

	All cohort (*n* = 178)		*APOE* ε *4* carriers (*n* = 65)		*APOE* ε *4* non-carriers (*n* = 113)	
FF correlation with:	*r*	*p*	*r*	*p*	*r*	*p*
Age	-0.01	0.890	0.200	0.111	-0.113	0.235
Sex	0.001	0.992	-0.109	0.389	-0.053	0.580
Education (years)	-0.077	0.309	-0.067	0.597	-0.080	0.399
Overall cognition	-0.026	0.731	-0.159	0.206	0.029	0.763
Working memory	-0.053	0.480	-0.092	0.467	-0.039	0.680
Episodic memory	0.002	0.978	-0.069	0.586	0.035	0.716
Executive motor	-0.021	0.778	-0.067	0.598	0.001	0.994
Language	0.002	0.984	-0.159	0.206	0.08	0.399
CES-D	0.259	0.001	0.199	0.115	0.287	0.002

Values are Pearson correlation coefficients, and *p*-values. SCD, subjective cognitive decline; MFQ_FF, frequency of forgetting scale of the Memory Functioning Questionnaire; CES-D, Center for Epidemiologic Studies Depression Scale.

A significant interaction of *APOE* genotype with SCD on brain activation was found in the following ROIs: anterior cingulate [*F*(1,209) = 4.527, *p* = 0.003], inferior frontal [*F*(1,209) = 4.231, *p* = 0.041], middle frontal [*F*(1,209) = 3,959, *p* = 0.048], and superior frontal gyri [*F*(1,209) = 4.414, *p* = 0.037].

**FIGURE 2:**
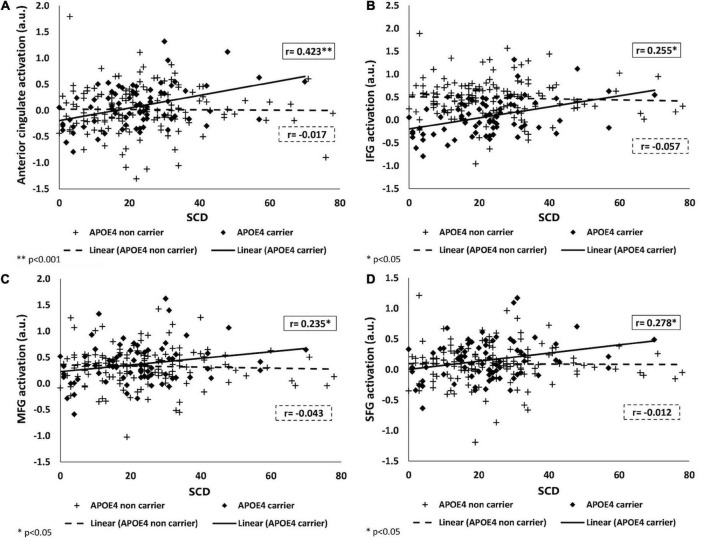
Associations of SCD with brain activation in *APOE* ε*4* carriers and non-carriers. Positive association between BOLD signal intensity during a working memory task and SCD among *APOE* ε*4* carriers (shown as black) and no association among *APOE* ε*4* non-carriers (shown as dashed-line) in the anterior cingulate **(A)**, IFG **(B)**, MFG **(C)**, and SFG **(D)**. SCD, subjective cognitive decline; IFG, MFG, SFG, inferior, middle, and superior frontal gyri; *APOE* ε*4*, apolipoprotein E, alleles ε34 or ε44.

To identify the source of the interactions, we examined the partial correlations between brain activation and SCD, stratified by *APOE* genotype, adjusting for the same covariates. For all significant interactions, among *APOE* ε*4* carriers, greater SCD was associated with more brain activation during a working memory task, while no association was found among *APOE* ε*4* non-carriers. There were no interactions of SCD with *APOE* on brain activation in the inferior parietal and middle temporal lobe. For all statistics values refer to Figure 2 and Table 3.

**TABLE 3:** Linear regression of *APOE* ε*4* genotype with SCD (MFQ_FF) on brain activation during a working memory task and on fractional anisotropy in the different brain regions adjusting for age, sex, and years of education.

	SCD		*APOE*		Interaction		Partial correlations							
							*APOE* ε *4* carriers				*APOE* ε *4* non-carriers			
**Brain activation during working memory task**														
**ROI**	***F*(1,209)**	***p***	***F*(1,209)**	***p***	***F*(1,209)**	***p***	***r***	***p***	***B***	**95% interval**	***r***	***p***	***B***	**95% interval**
Anterior cingulate	8.35	0.004	4.527	0.035	9.043	0.003	0.423	0.000	0.012	0.006–0.019	-0.017	0.845	0.000	−0.005 to 0.004
IFG	2.222	0.138	2.206	0.139	4.231	0.041	0.255	0.032	0.007	0.001–0.013	-0.057	0.507	-0.001	−0.006 to 0.003
MFG	2.474	0.117	1.998	0.159	3.959	0.048	0.235	0.049	0.007	0.000–0.013	-0.043	0.615	-0.001	−0.005 to 0.003
SFG	4.38	0.038	1.328	0.25	4.414	0.037	0.278	0.019	0.007	0.001–0.012	-0.012	0.893	0.000	−0.004 to 0.003
IPL	0.733	0.393	3.633	0.058	3.868	0.051	–	–	–	–	–	–	–	–
MTL	0.164	0.686	0.708	0.401	0.999	0.319	–	–	–	–	–	–	–	–
**Fractional anisotropy**														
**ROI**	***F*(1,199)**	***p***	***F*(1,199)**	***p***	***F*(1,199)**	***p***	***r***	***p***	***B***	**95% interval**	***r***	***p***	***B***	**95% interval**
Uncinate fasciculi	6.148	0.014	8.515	0.004	9.906	0.002	-0.369	0.002	-0.001	−0.001 to 0.000	0.057	0.517	0.000	0.000–0.000
Genu CC	0.095	0.759	0.039	0.843	0.006	0.941	–	–	–	–	–	–	–	–
Fornix	1.886	0.171	0.037	0.847	0.000	0.99	–	–	–	–	–	–	–	–
Cingulum	0.581	0.447	4.043	0.046	3.799	0.053	–	–	–	–	–	–	–	–
SLF	0.015	0.904	0.086	0.77	0.19	0.663	–	–	–	–	–	–	–	–

Partial correlations were examined only for regions presenting significant interactions; analyses are adjusted for main effects, age, sex, and years of education. SCD, subjective cognitive decline; MFQ_FF, frequency of forgetting scale of the Memory Functioning Questionnaire; IFG, MFG, SFG, inferior, middle, and superior frontal Gyri; IPL, inferior parietal lobe; MTL, middle temporal lobe; *APOE* ε*4*, apolipoprotein E, alleles ε34 or ε44; FA, fractional anisotropy; Genu CC, genu of the corpus callosum; SLF, superior longitudinal fasciculus.

We also found an interaction of *APOE* genotype with SCD on FA values, in the uncinate fasciculi [*F*(1,199) = 9.906, *p* = 0.002].

**FIGURE 3:**
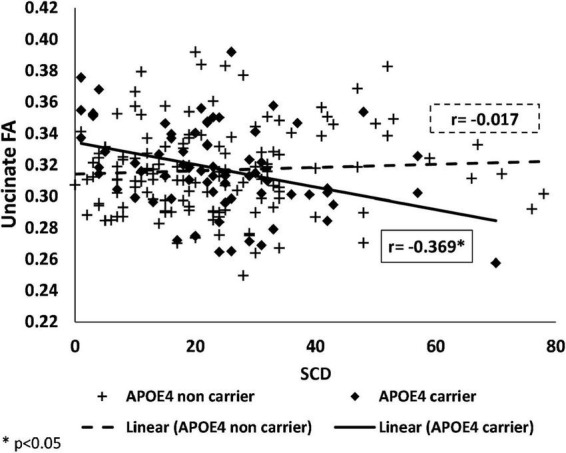
Associations of SCD with fractional anisotropy in *APOE* ε*4* carriers and non-carriers. Negative association between FA and SCD among *APOE* ε*4* carriers (shown as black) and no association among *APOE* ε*4* non-carriers (shown as a dashed-line) in the uncinate fasciculi. SCD, subjective cognitive decline; *APOE* ε*4*, apolipoprotein E, alleles ε34 or ε44; FA, fractional anisotropy.

Similarly to the brain activation results, in the stratified analysis, among *APOE* ε*4* carriers, higher SCD was associated with lower FA, whereas, no association was found in the *APOE* ε*4* non-carriers (see Figure 3 and Table 3). No significant interactions were found between SCD and *APOE* on the FA values of the genu of the corpus callosum, fornix, cingulum, superior longitudinal fasciculus, or on the MD values of all selected ROIs (Table 3 and Supplementary Table 1).

When using the total score of the MFQ as the SCD measure, only the uncinate displayed a significant interaction of *APOE* genotype with matter integrity [*F*(1,198) = 5.649, *p* = 0.018, see Supplementary Table 2].

Results remained essentially unchanged after adding depression scores, episodic memory, and executive function to the GLM models (see Supplementary Table 3).

In addition, after excluding one random sibling from each of the 15 pairs of siblings, results remained significant in all ROIs, except for the interactions of SCD with APOE on the Nback activation in the superior and middle frontal gyri, and middle temporal lobe which were attenuated {[*F*(1,194) = 3.827, *p* = 0.052], [*F*(1,194) = 3.380, *p* = 0.068], [*F*(1,194) = 0.916, *p* = 0.340] respectively, see Supplementary Table 4}.

Finally, after excluding participants with APOE e2/e4 alleles results remained significant in all ROIs, except the interactions of SCD with APOE on the Nback activation in the middle temporal lobe [*F*(1,202) = 0.632, *p* = 0.428, see Supplementary Table 5]. All secondary analyses results can be found in Supplementary Tables 1–5.

## Discussion

In this study, we used a comprehensive approach to examine the impact of SCD and moderation by *APOE* on structural and functional neural correlates that may underlie early memory dysfunction. We found significant interactions between SCD and *APOE* on both brain activation and white matter integrity. Specifically, the interaction of *APOE* genotype with SCD on brain activation was found in the anterior cingulate cortex and in the inferior, middle and superior frontal cortices, such that higher SCD was associated with increased brain activation in *APOE* ε*4* carriers. We also found a significant interaction of *APOE* genotype with SCD on white matter integrity in the uncinate fasciculi, where higher SCD was associated with lower FA specifically in *APOE* ε*4* carriers. Our results suggest that among individuals who are enriched for AD risk, *APOE* genotype modulates the association between SCD and brain alterations, possibly reflecting a preclinical disease process that precedes overt clinically significant cognitive dysfunction. Our study innovates by identifying APOE ε4 as a crucial modifying factor of structural and functional brain integrity in high risk individuals due to SCD and a parental history of AD.

Moreover, our results lend new evidence to the field by shedding light on potential abnormalities in neural activity during working memory observed in SCD. Working memory and other executive functions decline in individuals has been shown in SCD (Viviano et al., 2019) and characterizes cognitive functioning of AD patients (Kirova et al., 2015). Prior studies have largely focused on episodic memory tasks, but SCD may represent abnormalities in cognitive functions apart from episodic memory. Indeed, we found widespread differences in functional activation among brain regions that subserve working memory, a fundamental cognitive function underlying higher-order cognitive abilities, and which deteriorates early in the progression of AD (Baudic et al., 2006; Kirova et al., 2015).

In older adults, SCD has been found to be correlated with a decrease in overall gray matter volume (Saykin et al., 2006), in temporal areas known to be affected in AD (Schultz et al., 2015; Ryu et al., 2017). Regarding brain function, altered task-related brain activations have been associated with SCD (Rodda et al., 2009; Dumas et al., 2013). Similarly to our finding of higher activation in the frontal and anterior cingulate among *APOE* ε*4* carriers with SCD, increased activation in the prefrontal cortex among people with SCD has been found in previous studies (Rodda et al., 2009). Dumas et al. (2013) reported greater activation in a broad network of areas involved in a working memory task including the middle frontal gyrus, the precuneus and the cingulate gyrus in middle-aged adults reporting SCD (Dumas et al., 2013). Similarly, MCI and AD patients showed more activation in anterior cingulate, fusiform, and frontal and middle temporal areas while performing the working memory task (Yetkin et al., 2006).

The prefrontal cortex and anterior cingulate are well-known regions recruited during working memory tasks, and are also known to show greater activation as cognitive effort increases (Owen et al., 2005).

The *APOE* ε*4* allele has been associated with higher activation in several brain regions (Bookheimer et al., 2000) relative to non-carriers. Congruent with these findings linked to *APOE* ε*4*, we found increased brain activity in two of the areas that have previously presented reduced gray matter volume in patients with *APOE* ε*4* (Lee et al., 2016), the anterior cingulum and middle frontal gyrus. Thus, we speculate that the higher activation observed in APOE ε4 carriers may represent a compensatory process, by which participants who report SCD require the use of additional neural resources to achieve cognitive performance at normal levels (Bondi et al., 2005). This is consistent with “brain or cognitive reserve theory” defined as the ability to optimize performance through differential recruitment of brain networks (Stern, 2002) and is consistent with increased activation as a compensatory mechanism found in other conditions such as MCI (Solé-Padullés et al., 2009) and multiple sclerosis (Forn et al., 2007).

We have found that in *APOE* ε*4* carriers, SCD is associated with lower white matter integrity of the uncinate fasciculi. Reduced white matter integrity, is believed to reflect disruption of axonal structure or myelin loss (Basser, 1995), and has been previously described in MCI and AD (Alves et al., 2012), and among individuals at risk for AD due to *APOE* ε*4* genotype (Lee et al., 2016).

Supporting our findings of an association between SCD and white matter integrity, older adults with SCD have previously been observed to exhibit lower FA in the hippocampal body and entorhinal white matter (Ryu et al., 2017). Higher SCD has also been previously associated with lower white matter volume among *APOE* ε*4* carriers only (Dauphinot et al., 2020).

Impairment of FA in the uncinate fasciculi in high AD risk individuals is reinforced by another study from our group showing that FH participants who were also *APOE* ε*4* carriers, had the lowest FA compared to other groups (Bendlin et al., 2010). The uncinate fasciculus, a cortico-cortical association tract, supports decision-making, emotion, and episodic memory, and exhibits changes during the pathophysiology of AD (Kier et al., 2004). There is evidence that a disruption of the uncinate fasciculus fibers can cause severe memory impairment (Levine et al., 1998). We suggest that high SCD accompanied by low white matter integrity in this tract may represent an early indicator of incipient cognitive impairment, though further longitudinal studies should be conducted.

While the structural and functional data in this study were assessed in independent models, the use of the two imaging modalities may shed additional light on structure and function relationship. According to disconnection theories, reduced white matter integrity can be related to changes in cortical activation mediating cognitive function (Bartzokis et al., 2004). Given that the white matter integrity of the uncinate fasciculi, a fronto-temporal association tract, was found to be impaired in people with higher SCD, it is plausible that frontal areas which are connected through this tract exhibit higher activation as a compensatory response.

Study strengths include the relatively large cohort of middle-aged participants enriched for AD risk due to a parental family history who had both structural and functional MRI; the use of a well-validated questionnaire assessing broad aspects of SCD rather than the often used 1-question for SCD (Hong et al., 2015; Schultz et al., 2015) as well as comprehensive neuroimaging to facilitate testing the association between SCD and both structural and functional brain measures, as well as moderating impacts of genotype. Our study is limited by its cross sectional design. The IRAP study longitudinal component is ongoing and examination of the inter-relationship of SCD with *APOE* ε*4* on objectively measured cognitive decline is warranted. The IRAP study is based on a community sample of individuals with FH, and results may not generalize to individuals without a FH. The choice of ROIs in our study was done *a priori* and therefore all primary analyses were not adjusted for multiple comparisons. The interaction between *APOE* genotype and brain activation in the anterior cingulate and white matter integrity in the uncinate remained significant after applying Bonferroni correction of 0.05/6 = 0.008 for the fMRI analysis and 0.05/5 = 0.01 for the DTI analysis. Finally, our white-matter integrity methods are limited by a relatively small number of directions (32 directions) and due to the fact that FA and MD values were extracted from ROIs rather than from probabilistic tractography.

## Conclusion

We conclude that *APOE* ε*4* allele moderates the association between SCD and neural correlates supporting memory function. Our results indicate that among cognitively normal, middle-aged individuals due to a family history of AD and the *APOE* ε*4* allele, SCD is associated with brain alteration including disrupted white matter integrity and abnormal brain activity during a working memory task. It is possible that these neural alterations reflect incipient AD pathology in a phase where objective cognitive impairment is yet undetectable. AD offspring with *APOE* ε*4* presenting SCD may represent an enriched population and serve as potential candidates for future clinical trials of interventions for prevention of AD. In addition, our results suggest that clinicians may need to follow particularly closely those asymptomatic individuals who are enriched for AD risk and are reporting SCD, as it may represent a window for intervention before frank AD dementia develops.

## Data availability statement

The raw data supporting the conclusions of this article will be made available by the authors, without undue reservation.

## Ethics statement

The studies involving human participants were reviewed and approved by the Sheba Medical Center Institutional Review Board (IRB). The patients/participants provided their written informed consent to participate in this study.

## Author contributions

LB-A researched the data and wrote the manuscript. RR-S and MS researched the data, reviewed the manuscript, and contributed to the discussion. GT, RR, and LG researched the data and reviewed the manuscript. AS and IS-G researched the data. BB, EO, and AH reviewed the manuscript and contributed to the discussion. AL researched the data and wrote and reviewed the manuscript. All authors contributed to the article and approved the submitted version.

## Footnotes

1
https://www.lgcgroup.com/
