# Supplementary material for: Neural correlates of subjective cognitive decline in adults at high risk for Alzheimer’s disease
Source: Front Aging Neurosci. 2023 Feb 1;15:1067196. doi: 10.3389/fnagi.2023.1067196 (PMC9930909; doi:10.3389/fnagi.2023.1067196)
Supplement: Supplementary file 1 [file Table_1.DOCX]

***Supplementary Tables***

***Table 1***– *linear regression of APOE* ε4 *genotype with SCD (MFQ_FF) on* ***MD*** *(mean diffusivity) in the different brain regions adjusting for age, sex and years of education*

|  | *SCD* | | *APOE* | | *Interaction* | |
| --- | --- | --- | --- | --- | --- | --- |
|  |  |  |  |  |  |  |
|  |  |  |  |  |  |  |
| *Mean Diffusivity* | | | | | | |
| *ROI* | *F(1,197)* | *p* | *F(1,197)* | *p* | *F(1,197)* | *p* |
| **Uncinate Fasciculi** | 0.876 | 0.351 | 1.613 | 0.206 | 1.914 | 0.168 |
| **Genu CC** | 1.970 | 0.162 | 1.204 | 0.274 | 0.028 | 0.867 |
| **Fornix** | 2.640 | 0.106 | 0.006 | 0.936 | 0.014 | 0.906 |
| **Cingulum** | 0.548 | 0.460 | 0.720 | 0.397 | 0.317 | 0.574 |
| **SLF** | 0.526 | 0.469 | 0.933 | 0.335 | 0.180 | 0.672 |

*Analyses are adjusted for main effects, age, sex and years of education; SCD = subjective cognitive decline; MFQ_FF = Frequency of forgetting scale of the Memory Functioning Questionnaire; APOE* ε4 *= Apolipoprotein E, alleles ε34 or ε44; MD=* *mean diffusivity; Genu CC= Genu of the corpus callosum; SLF= superior longitudinal fasciculus.*

***Table 2***– *linear regression of APOE* ε4 *genotype with SCD (MFQ Total) on brain activation during a working memory task and on fractional anisotropy in the different brain regions adjusting for age, sex and years of education*

|  | *SCD* | | *APOE* | | *Interaction* | | *partial correlations* | | | |
| --- | --- | --- | --- | --- | --- | --- | --- | --- | --- | --- |
|  |  |  |  |  |  |  |  |  |  |  |
|  |  |  |  |  |  |  | *APOE* ε4 *carriers* | | *APOE* ε4 *non carriers* | |
| ***Brain activation during working memory task*** | | | | | | | | | | |
| *ROI* | *F(1,207)* | *p* | *F(1,207)* | *p* | *F(1,207)* | *p* | *r* | *p* | *r* | *p* |
| **Anterior Cingulate** | 4.655 | 0.032 | 3.190 | 0.076 | 2.713 | 0.101 | - | - | - | - |
| **IFG** | 0.496 | 0.482 | 1.284 | 0.258 | 1.103 | 0.295 | - | - | - | - |
| **MFG** | 0.638 | 0.425 | 0.932 | 0.335 | 0.764 | 0.383 | - | - | - | - |
| **SFG** | 1.022 | 0.313 | 1.698 | 0.194 | 1.249 | 0.265 | - | - | - | - |
| **IPL** | 0.066 | 0.798 | 0.622 | 0.431 | 0.691 | 0.407 | - | - | - | - |
| **MTL** | 0.048 | 0.827 | 1.562 | 0.213 | 1.543 | 0.216 | - | - | - | - |
| ***Fractional Anisotropy*** | | | | | | | | | | |
| *ROI* | *F(1,198)* | *p* | *F(1,198)* | *p* | *F(1,198)* | *p* | *r* | *p* | *r* | *p* |
| **Uncinate Fasciculi** | 3.350 | 0.069 | 5.649 | 0.018 | 6.063 | 0.015 | 0.314 | 0.010 | -0.05 | 0.566 |
| **Genu CC** | 0.026 | 0.873 | 0.235 | 0.629 | 0.165 | 0.685 | - | - | - | - |
| **Fornix** | 1.724 | 0.191 | 0.022 | 0.883 | 0.012 | 0.912 | - | - | - | - |
| **Cingulum** | 0.976 | 0.324 | 0.572 | 0.450 | 0.749 | 0.388 | - | - | - | - |
| **SLF** | 0.000 | 0.991 | 0.203 | 0.653 | 0.178 | 0.673 | - | - | - | - |

*Partial correlations were examined only for regions presenting significant interactions; Analyses are adjusted for main effects, age, sex and years of education;*

*SCD = subjective cognitive decline; MFQ = Memory Functioning Questionnaire; APOE ε4 = Apolipoprotein E, alleles ε34 or ε44; IFG, MFG, SFG = Inferior, middle and superior frontal Gyri; IPL= inferior parietal lobe; MTL= Middle temporal lobe; FA=fractional anisotropy; Genu CC= Genu of the corpus callosum; SLF= superior longitudinal fasciculus.*

***Table 3 -***  *Secondary analyses applied to regions which showed significant interaction of the APOE genotype with SCD (MFQ_FF) on brain activation during a working memory task and on fractional anisotropy in the primary analyses, adjusting for depressive symptoms measured by CES-D (Model 1), for episodic memory (Model 2) and for executive function (Model 3) in addition to age, sex and years of education.*

|  | *Model 1 - Interaction with* ***depressive symptoms***  *as covariate* | | *Model 2 -*  *Interaction with* ***episodic memory***  *as covariate* | | *Model 3 -*  *Interaction with* ***executive function***  *as covariate* | |
| --- | --- | --- | --- | --- | --- | --- |
|  |  |  |  |  |  |  |
|  |  |  |  |  |  |  |
| ***Brain activation - SCD*** | | | | | | |
| *ROI* | *F(1,202)* | *p* | *F(1,208)* | *p* | *F(1,208)* | *p* |
| **Anterior Cingulate** | 9.343 | 0.003 | 8.979 | 0.003 | 8.946 | 0.003 |
| **IFG** | 4.792 | 0.030 | 4.155 | 0.043 | 4.356 | 0.038 |
| **MFG** | 4.723 | 0.031 | 3.862 | 0.051 | 4.093 | 0.044 |
| **SFG** | 5.057 | 0.026 | 4.326 | 0.039 | 4.592 | 0.033 |
| ***FA - SCD*** | | | | | | |
|  | *F(1,202)* | *p* | *F(1,198)* | *p* | *F(1,198)* | *p* |
| **Uncinate Fasciculi** | 9.472 | 0.002 | 9.874 | 0.002 | 10.986 | 0.001 |

*SCD=subjective cognitive decline; MFQ_FF = Frequency of forgetting scale of the Memory Functioning Questionnaire; IFG,MFG,SFG=Inferior, Medial and Superior Frontal Gyri,*

*FA=fractional anisotropy; CES-D = Center for Epidemiologic Studies Depression Scale.*

***Table 4***– *linear regression of APOE* ε4 *genotype with SCD (MFQ_FF) on brain activation during a working memory task and on fractional anisotropy ,* *excluding one random sibling from each pair in the different brain regions adjusting for age, sex and years of education*

|  | *SCD* | | *APOE* | | *Interaction* | | *partial correlations* | | | |
| --- | --- | --- | --- | --- | --- | --- | --- | --- | --- | --- |
|  |  |  |  |  |  |  |  |  |  |  |
|  |  |  |  |  |  |  | *APOE* ε4 *carriers* | | *APOE* ε4 *non carriers* | |
| ***Brain activation during working memory task*** | | | | | | | | | | |
| *ROI* | *F(1,194)* | *p* | *F(1,194)* | *p* | *F(1,194)* | *p* | *r* | *p* | *r* | *p* |
| **Anterior Cingulate** | 9.816 | 0.002 | 5.213 | 0.024 | 10.057 | 0.002 | 0.460 | 0.000 | -0.012 | 0.890 |
| **IFG** | 2.218 | 0.138 | 2.434 | 0.120 | 4.115 | 0.044 | 0.255 | 0.036 | -0.056 | 0.533 |
| **MFG** | 3.287 | 0.071 | 1.673 | 0.197 | 3.380 | 0.068 | - | - | - | - |
| **SFG** | 5.326 | 0.022 | 1.127 | 0.290 | 3.827 | 0.052 | - | - | - | - |
| **IPL** | 1.980 | 0.161 | 3.300 | 0.071 | 3.532 | 0.062 | - | - | - | - |
| **MTL** | 0.147 | 0.702 | 0.642 | 0.424 | 0.916 | 0.340 | - | - | - | - |
| ***Fractional Anisotropy*** | | | | | | | | | | |
| *ROI* | *F(1,183)* | *p* | *F(1,183)* | *p* | *F(1,183)* | *p* | *r* | *p* | *r* | *p* |
| **Uncinate Fasciculi** | 6.550 | 0.011 | 7.513 | 0.007 | 7.783 | 0.006 | -0.363 | 0.003 | 0.021 | 0.819 |
| **Genu CC** | 0.013 | 0.909 | 0.359 | 0.550 | 0.137 | 0.712 | - | - | - | - |
| **Fornix** | 0.639 | 0.425 | 0.229 | 0.633 | 0.007 | 0.932 | - | - | - | - |
| **Cingulum** | 0.725 | 0.396 | 2.760 | 0.098 | 2.405 | 0.123 | - | - | - | - |
| **SLF** | 0.022 | 0.882 | 0.159 | 0.691 | 0.218 | 0.641 | - | - | - | - |

*Partial correlations were examined only for regions presenting significant interactions; Analyses are adjusted for main effects, age, sex and years of education;*

*SCD = subjective cognitive decline; MFQ_FF = Frequency of forgetting scale of the Memory Functioning Questionnaire; IFG, MFG, SFG = Inferior, middle and superior frontal Gyri; IPL= inferior parietal lobe; MTL= Middle temporal lobe; APOE ε4 = Apolipoprotein E, alleles ε34 or ε44; FA=fractional anisotropy; Genu CC= Genu of the corpus callosum; SLF= superior longitudinal fasciculus.*

***Table 5***– *linear regression of APOE* ε4 *genotype with SCD (MFQ_FF) on brain activation during a working memory task and on fractional anisotropy ,excluding APOE e2/e4 carriers in the different brain regions adjusting for age, sex and years of education*

|  | *SCD* | | *APOE* | | *Interaction* | | *partial correlations* | | | |
| --- | --- | --- | --- | --- | --- | --- | --- | --- | --- | --- |
|  |  |  |  |  |  |  |  |  |  |  |
|  |  |  |  |  |  |  | *APOE* ε4 *carriers* | | *APOE* ε4 *non carriers* | |
| ***Brain activation during working memory task*** | | | | | | | | | | |
| *ROI* | *F(1,202)* | *p* | *F(1,202)* | *p* | *F(1,202)* | *p* | *r* | *p* | *r* | *p* |
| **Anterior Cingulate** | 8.803 | 0.003 | 4.468 | 0.036 | 9.730 | 0.002 | 0.459 | 0.000 | -0.017 | 0.845 |
| **IFG** | 2.384 | 0.124 | 1.923 | 0.167 | 4.549 | 0.034 | 0.281 | 0.025 | -0.057 | 0.507 |
| **MFG** | 3.307 | 0.07 | 2.524 | 0.114 | 5.089 | 0.025 | 0.284 | 0.023 | -0.043 | 0.615 |
| **SFG** | 5.213 | 0.023 | 1.693 | 0.195 | 5.396 | 0.021 | 0.322 | 0.010 | -0.012 | 0.893 |
| **IPL** | 1.894 | 0.170 | 5.358 | 0.022 | 6.276 | 0.013 | 0.299 | 0.016 | -0.091 | 0.287 |
| **MTL** | 0.338 | 0.562 | 0.311 | 0.578 | 0.632 | 0.428 | - | - | - | - |
| ***Fractional Anisotropy*** | | | | | | | | | | |
| *ROI* | *F(1,193)* | *p* | *F(1,193)* | *p* | *F(1,193)* | *p* | *r* | *p* | *r* | *p* |
| **Uncinate Fasciculi** | 7.152 | 0.008 | 9.486 | 0.002 | 11.071 | 0.001 | -0.400 | 0.001 | 0.057 | 0.517 |
| **Genu CC** | 0.064 | 0.801 | 0.003 | 0.956 | 0.009 | 0.925 | - | - | - | - |
| **Fornix** | 1.260 | 0.263 | 0.050 | 0.823 | 0.047 | 0.829 | - | - | - | - |
| **Cingulum** | 0.439 | 0.508 | 3.695 | 0.056 | 3.261 | 0.072 | - | - | - | - |
| **SLF** | 0.011 | 0.916 | 0.080 | 0.777 | 0.147 | 0.702 | - | - | - | - |

*Partial correlations were examined only for regions presenting significant interactions; Analyses are adjusted for main effects, age, sex and years of education; SCD = subjective cognitive decline; MFQ_FF = Frequency of forgetting scale of the Memory Functioning Questionnaire; IFG, MFG, SFG = Inferior, middle and superior frontal Gyri; IPL= inferior parietal lobe; MTL= Middle temporal lobe; APOE ε4 = Apolipoprotein E, alleles ε34 or ε44; FA=fractional anisotropy; Genu CC= Genu of the corpus callosum; SLF= superior longitudinal fasciculus.*
